# HealthData@MAD-R&I: Protocol for Design and Development of a Regional Health Data Infrastructure to Enable Secondary Use of Health Data in Research and Innovation

**DOI:** 10.2196/82815

**Published:** 2026-03-20

**Authors:** Montserrat León-García, Sergio Álvarez-Pérez, Janire Gesto-Gómez, Clara Urbano-Molina, Sonia Soto-Díaz, Juan Cárdenas-Valladolid, Luis Rodríguez-Rodríguez, Antonio Díaz-Holgado, Isabel del Cura-González, Javier De La Cruz-Bertolo, Noelia García-Barrio, Juan Luis Cruz-Bermúdez, Cristina García-Fernández, Carlos Rodríguez-Antolín, Laila García-Aldars, Elsa María Moreda-Sánchez, Álvaro Roldán López, José María Veganzones Alonso-Cortés, Ana Isabel Gonzalez Gonzalez, Miguel A Salinero-Fort, HealthData@MAD-R&I Working Group

**Affiliations:** 1 Fundación para la Investigación e Innovación Biosanitaria de Atención Primaria (FIIBAP) Madrid, Madrid Spain; 2 Dirección General de Investigación y Docencia Consejería de Sanidad de la Comunidad de Madrid Madrid, Madrid Spain; 3 Universidad Alfonso X el Sabio Madrid, Madrid Spain; 4 Frailty, Multimorbidity Patterns and Mortality in the Elderly Population Residing in the Community Hospital La Paz Institute for Health Research (IdiPAZ) Madrid, Madrid Spain; 5 Grupo de Patología Musculoesquelética Fundación para la Investigación Biomédica del Hospital Clínico San Carlos Instituto de Investigación Sanitaria San Carlos (IdISSC) Madrid, Madrid Spain; 6 Gerencia Asistencial de Atención Primaria Servicio Madrileño de Salud Consejería de Sanidad de la Comunidad de Madrid Madrid, Madrid Spain; 7 Departamento de Especialidades Médicas y Salud Pública Universidad Rey Juan Carlos Madrid, Madrid Spain; 8 Network for Research on Chronic Diseases, Primary Care, and Health Promotion (RICAPPS) Madrid, Madrid Spain; 9 Instituto de Investigación Sanitaria Gregorio Marañón Madrid, Madrid Spain; 10 Ageing research center Karolinska Institute Solna, Stockholm Sweden; 11 Grupo de Investigación e Innovación en Transformación Digital e Ingeniería Biomédica Hospital Universitario 12 de Octubre Instituto de Investigación Sanitaria Hospital 12 de Octubre (imas12) Madrid, Madrid Spain; 12 Dirección General de Salud Digital Consejería de Digitalización de la Comunidad de Madrid Madrid, Madrid Spain; 13 Instituto de Genética Médica y Molecular Instituto de Investigación Sanitaria del Hospital Universitario La Paz Madrid, Madrid Spain; 14 See Acknowledgments

**Keywords:** secondary use, interoperability, digital health, advanced predictive models, European Health Data Space, federated data, clinical research, innovation, public health

## Abstract

**Background:**

The exponential growth of electronic health records (EHRs), together with the recent entry into force of the European Health Data Space (EHDS) Regulation, highlights the urgent need for secure, interoperable environments that support the secondary use of health data. In response, HealthData@MAD-R&I emerges as a pioneering initiative in Madrid (Spain), aligned with the EHDS strategy and the European Commission’s vision for data sovereignty and trustworthy data reuse.

**Objective:**

This study aims to design and implement HealthData@MAD-R&I, a regional health data space that enables responsible access to high-quality health data to support clinical research, health care innovation, and evidence-informed decision-making.

**Methods:**

HealthData@MAD-R&I aims to establish an ethically governed, scalable, and sustainable health data space. The project adopts a structured, iterative methodology based on the Data Management Association (DAMA) framework. and it is organized into 9 work packages across three thematic areas: (1) project management and sustainability, (2) governance and technological infrastructure, and (3) validation through 4 real-world use cases. The technical architecture adopts a hybrid federated model built with open-source components, and data harmonization is performed using the Observational Medical Outcomes Partnership (OMOP) common data model to ensure semantic and syntactic interoperability. Artificial intelligence, machine learning, natural language processing, and privacy-preserving techniques are applied for data curation and secure access.

**Results:**

As of November 2025, the main achievements include (1) the development of a data governance model that articulates principles of quality, transparency, and regulatory compliance; (2) the design of a secure, interoperable technological architecture with federated capabilities based on international standards (DAMA and OMOP); and (3) the implementation of 4 use cases—optimizing rheumatology referrals, characterizing care pathways for long-term survivors of breast cancer, predicting unplanned hospitalizations, and evaluating the effectiveness of statins in older adults—to validate the data space while addressing diverse clinical and policy challenges. Together, these components demonstrate the potential of regional data spaces to support evidence-based clinical practice and public policy.

**Conclusions:**

HealthData@MAD-R&I seeks to strengthen Madrid’s role in digital health innovation and contribute to the broader European health data ecosystem by promoting interoperable, privacy-compliant secondary use of health data. The project’s evaluation framework includes indicators for data quality, research outputs, and health care system impact.

**International Registered Report Identifier (IRRID):**

DERR1-10.2196/82815

## Introduction

The exponential growth of electronic health records (EHRs) and other digital health data sources has created unprecedented opportunities to improve health care delivery, foster scientific discovery, and support evidence-informed policymaking [1-7]. The COVID-19 pandemic further underscored the need for timely, high-quality health data to support public health responses and accelerate research [5]. In this context, the European Commission launched the European Health Data Space (EHDS) strategy in 2022, aiming to establish a unified health data market that enhances Europe’s competitiveness, promotes data sovereignty, and supports the secure and ethical reuse of health data across borders and sectors [3-10]. The publication of the EHDS regulation in the *Official Journal of the European Union* on March 5, 2025, marked a key milestone in the transition from strategy to implementation [11].

Health data spaces are central to this vision, serving as the foundational infrastructure for secure, interoperable, and trustworthy access to health data across member states. They have the potential to enhance population health by enabling large-scale research, improving disease prevention and treatment, advancing precision medicine, and supporting more efficient health system management [4,6,7]. A key component of this paradigm is the secondary use of health data—information originally collected during routine clinical care and subsequently repurposed for research, innovation, or policymaking. Despite its promise, secondary use remains limited by persistent challenges related to interoperability, governance, data quality, privacy protection, and ethical oversight [12-16].

HealthData@MAD-R&I emerges in response to these challenges as a regional initiative in Madrid, Spain, designed to develop a federated, secure, and ethically governed health data space for the secondary use of health data. The project is funded by the Ministry for Digital Transformation and Civil Service and by the European Union through the European Recovery Instrument (“Next Generation EU”), under Spain’s Recovery, Transformation, and Resilience Plan. It builds upon existing digital health infrastructure in the region, including the Infobanco project [3] and the Hipócrates secondary-use platform, which integrates large-scale clinical data from multiple providers. The initiative brings together health care institutions, research centers, technology companies, and public authorities within a collaborative ecosystem aimed at enhancing the accessibility, usability, and trustworthiness of health data [3,4,6,12-16].

The objective of this project is to design and implement HealthData@MAD-R&I, a regional health data space that enables the secure and responsible secondary use of health data to support research, innovation, and evidence-informed policymaking, in alignment with European and national data governance frameworks. This manuscript presents the implementation protocol for the development of this infrastructure, with a focus on governance, interoperability, and validation through real-world use cases as part of a systems-level digital health innovation initiative.

## Methods

### Overview

**Figure 1:**
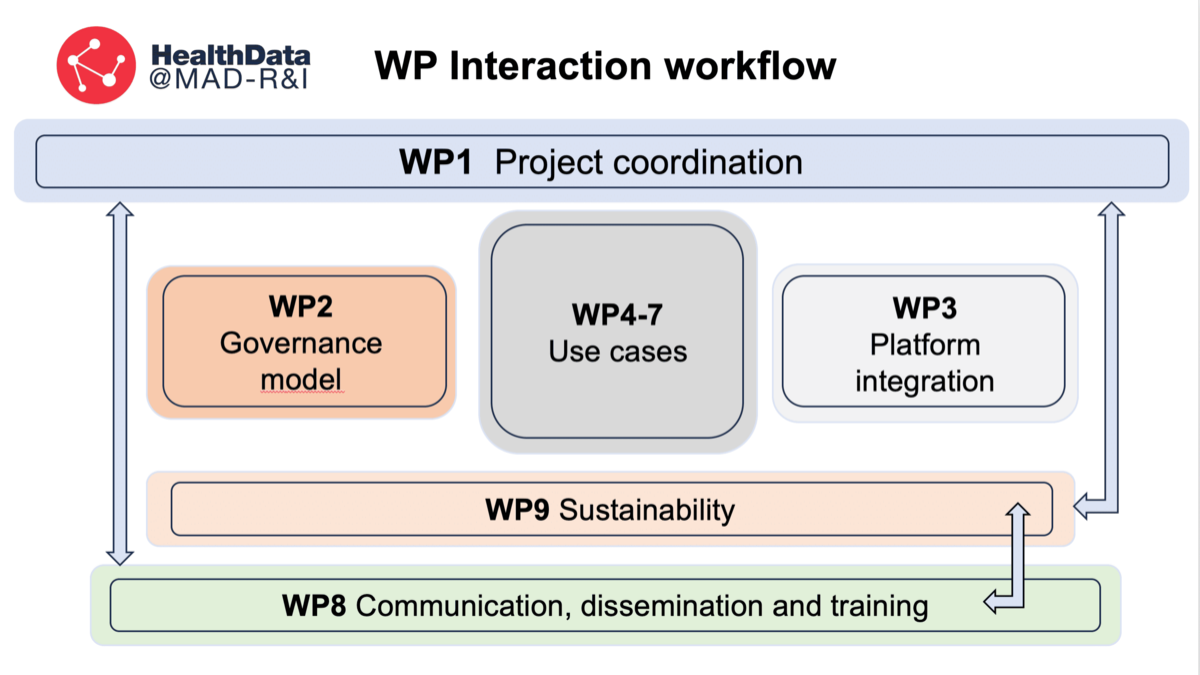
Work packages interaction flow. WP: work package.

The implementation of HealthData@MAD-R&I follows a structured, phased methodology articulated through 9 interconnected work packages (WP1-WP9), each addressing a critical dimension of the project to ensure effective management, governance, security, communication, and long-term scalability (Table 1 and Figure 1). This comprehensive approach integrates data governance, technological infrastructure, and scientific validation while ensuring full compliance with ethical principles and both national [17] and European regulatory frameworks [11].

Figure 1 shows how the 9 WPs of HealthData@MAD-R&I interact with each other, highlighting dependencies, information flows, and oversight. It clarifies roles, reduces perceived overlap, and shows how coordination, governance, technology, communication, and sustainability are connected throughout the project life cycle.

The system architecture is based on a hybrid federated data-space model built using open-source components, which enables flexibility, scalability, and interoperability across institutions. The methodological approach is aligned with the Data Management Association (DAMA) framework [18] and incorporates best practices in data governance, standardization, and advanced analytics, including artificial intelligence (AI), machine learning (ML), and natural language processing (NLP). These elements collectively support the development of a secure, standardized, and analytically robust environment for secondary use of health data.

To streamline execution and ensure coherence across diverse activities, the 9 WPs are organized into three thematic areas: (1) core operational pillars, comprising project management, communication, professional training, and long-term scalability and sustainability; (2) governance and technical architecture, addressing regulatory alignment, data management rules, and the design of the secure processing infrastructure; and (3) validation through real-world use cases, which assess the functionality, clinical relevance, and public-health impact of the data space.

**Table 1:** Overview of the structure of the HealthData@MAD-R&I project.

Thematic areas	Work packages (WPs)
A1. Core aspects of the project	WP1. Project management WP8. Communication and professional training. WP9. Long-term scalability and sustainability and scalability of the data space
A2. Design and architecture of the data space	WP2. Development of a robust data governance framework WP3. Technical architecture of the data space and data curation processes
A3. Validation of the data space through real-world use cases	WP4. Use case number 1: optimization of referral pathways for patients with rheumatic and musculoskeletal diseases WP5. Use case number 2: longitudinal analysis of care pathways for women who are long-term survivors of breast cancer WP6. Use case number 3: development of a predictive model for unplanned hospitalizations WP7. Use case number 4: evaluation of the effectiveness of statins in the primary prevention of cardiovascular events among individuals aged 75 years and older

### Area 1: Core Aspects of the Project: Management, Communication, Professional Training, and Scalability and Sustainability (WPs 1, 8, and 9)

This first thematic area addresses the foundational elements necessary for the successful deployment and long-term viability of the HealthData@MAD-R&I health data space. It encompasses project management, communication strategies, professional training, and sustainability planning. These components are operationalized through 3 highly intertwined WPs (WP1, WP8, and WP9).

WP1 focuses on project management, establishing the organizational structure, coordination mechanisms, and oversight procedures required to guide the implementation process. The management approach is grounded in the Agile methodology [19], which supports iterative planning, continuous evaluation, and stakeholder participation. This allows for flexible and efficient workflows, fostering collaboration among technical, scientific, and administrative teams. Key activities include the identification and mitigation of risks, the systematic monitoring of milestones and deliverables, and the facilitation of cross-functional coordination to ensure alignment across all components of the project.

A central objective of WP1 is to build and maintain public trust. To ensure meaningful citizen involvement, WP1 includes a structured public and stakeholder engagement plan consisting of (1) the establishment of a patient and citizen advisory board with representation from patient associations and community organizations; (2) periodic public consultations to gather feedback on governance, ethical frameworks, and data-access policies; and (3) open communication channels through the project website and the RIMASalud (institutional repository of the health department) portal for submitting questions, suggestions, or concerns. These mechanisms promote transparency, support co-creation of governance decisions, and ensure that societal expectations and values are reflected in the design and operation of the health data space.

WP8 addresses the project’s communication strategy and capacity-building activities. It sets out strategies for communicating project progress, raising stakeholder awareness, and improving digital health literacy, particularly regarding the purpose and value of secondary health data use. Communication activities target multiple audiences (the general public, health care professionals, researchers, and policymakers) and include scientific dissemination through publications and conference presentations, engagement through digital media, and outreach initiatives tailored to patients and citizens.

In parallel, the professional training component delivers workshops, seminars, and user guides designed for researchers, health care professionals, data scientists, and other relevant actors to foster capacity-building in the effective use of the data space. An evaluation framework will assess the impact of these efforts by measuring visibility, stakeholder engagement, user satisfaction, and knowledge acquisition.

WP9 ensures the long-term scalability and sustainability of HealthData@MAD-R&I beyond the initial implementation phase. It defines a roadmap for the progressive incorporation of new real-world use cases and for the continued evolution of the infrastructure. WP9 also guarantees strategic alignment with the EHDS, enabling future interoperability and supporting cross-border data exchange.

To guarantee sustainability, the project adopts a mixed economic model that combines regional public funding with cost-sharing agreements among participating institutions. In addition, a controlled data-access service fee will apply to external research requests—particularly from industry or nonaffiliated organizations—under transparent and equitable governance rules. WP9 also explores public-private partnerships under open-innovation frameworks, allowing private entities to contribute technical capabilities or infrastructure support while preserving public oversight and data safeguards.

A reinvestment mechanism will channel revenues from data-access services into continuous improvement activities, including infrastructure maintenance, cybersecurity reinforcement, quality-assurance processes, and ongoing capacity-building for professionals. Long-term operation of the platform will be supported through framework agreements with the General Directorate for Digital Health (DGSD) [17] and integration with future national and European digital health programs.

All documentation, technical specifications, and progress reports are published through the institutional repository of the health department (RIMASalud) and will continue to be updated as the infrastructure matures.

### Area 2: Design and Architecture of the HealthData@MAD-R&I Space: Governance and Technology (WPs 2 and 3)

The second thematic area focuses on the governance and technological foundations required to build a secure, interoperable, and ethically sound data space for the secondary use of health data. It encompasses the design and implementation of a governance model (WP2) and the technical architecture and data curation processes that underpin the infrastructure (WP3). Together, these WPs ensure that HealthData@MAD-R&I meets the highest standards of regulatory compliance, data quality, and operational functionality.

WP2 is focused on developing a robust data governance framework aligned with both national and European regulatory requirements [8,11,17]. The governance framework establishes the lawful basis for data processing as the performance of a task carried out in the public interest for health research and innovation, pursuant to Articles 6(1)(e) and 9(2)(j) of the General Data Protection Regulation (GDPR) [17]. A Data Protection Impact Assessment (DPIA) will be conducted during implementation, under the oversight of the Data Protection Officer of the Madrid Health Service (Regional Health System of Madrid (Servicio Madrileño de Salud [SERMAS]).

Processing activities will follow a controlled workflow with traceable audit logging, and data made available within the secure processing environment (SPE) will undergo privacy-preserving transformations—including pseudonymization, date shifting, suppression of quasi-identifiers, and aggregation of rare events—in accordance with ISO (International Organization for Standardization)/IEC (International Electrotechnical Commission) 20889:2018 [20,21]. Differential-privacy techniques will be applied to aggregated outputs.

Information security and confidentiality procedures are aligned with the National Security Framework (ENS) and ISO/IEC 27001 [20], ensuring compliance with Spanish and European cybersecurity requirements.

In alignment with GDPR [17] and the citizen rights provisions of the EHDS [8,11], the infrastructure will include a fully automated consent and opt-out management service integrated with institutional EHR systems. Patient choices will be securely stored as standardized Health Level Seven Fast Healthcare Interoperability Resources (HL7 FHIR) [22] and propagated across all participating institutions through FHIR-based interoperability services. Each processing request will query the consent registry in real time, ensuring that opt-out or withdrawal decisions are enforced consistently across the system. This mechanism guarantees traceability, preserves patient autonomy, and provides a practical and interoperable solution for system-wide compliance with data-participant rights.

The governance model also defines the roles and responsibilities of health data holders, health data access bodies, and data users across the lifecycle, following the principles of the DAMA framework [18], to promote responsible stewardship and institutional accountability.

**Figure 2:**
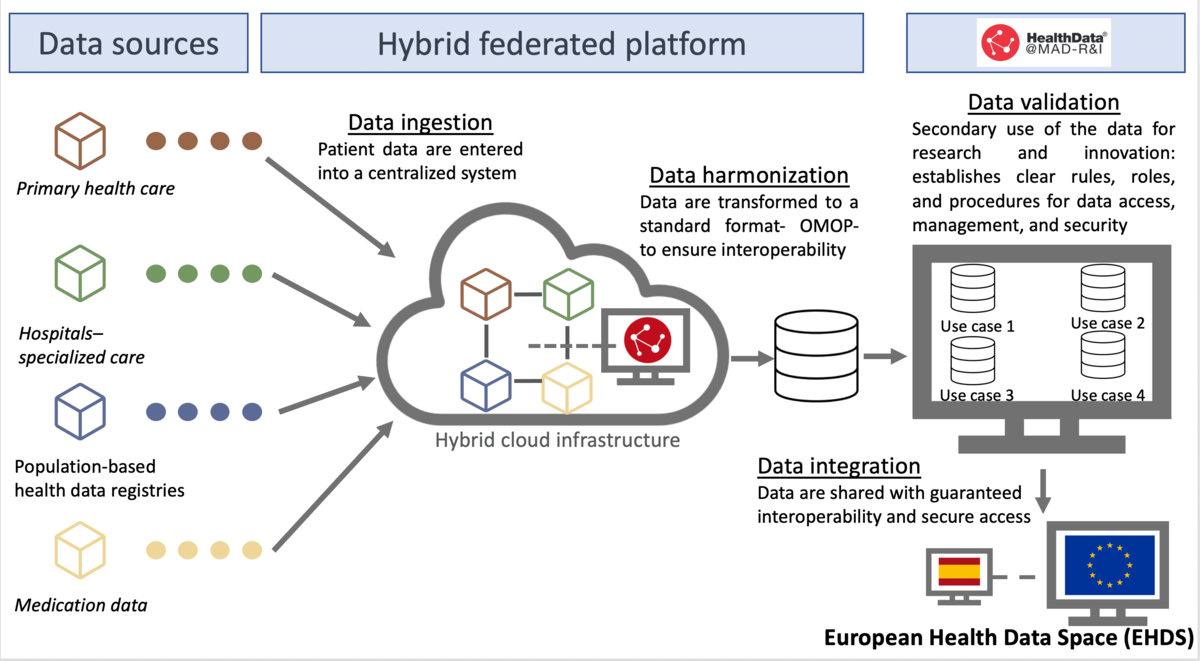
Architecture and governance structure of the HealthData@MAD-R&I infrastructure. OMOP: Observational Medical Outcomes Partnership.

WP3 addresses the technical architecture and data-curation processes that underpin the functionality, scalability, and interoperability of the data space (Figure 2). The architecture is built using open-source components and deployed through the Cloudera platform (Cloudera, Inc), under the supervision of the DGSD.

Figure 2, illustrates the three main layers: (1) the data layer, which integrates clinical and administrative datasets (eg, CMBD [Conjunto Mínimo Básico de Datos], SELENE, and FARMADRID) harmonized to the OMOP (Observational Medical Outcomes Partnership) common data model; (2) the platform layer, including the Federated Coordination Node, ETL and deidentification modules, metadata registry, and SPEs; and (3) the governance layer, encompassing access management, audit logging, and compliance oversight by the Directorate General for Research and Teaching and the Directorate General for Digital Health. Data flow proceeds from local institutional sources to standardized repositories through privacy-preserving pipelines, ensuring traceability, interoperability, and lawful reuse for research and innovation.

To ensure legal and technical interoperability across institutions with varying levels of data maturity, HealthData@MAD-R&I establishes a Federated Coordination Node (FCN) hosted within the regional cloud environment. The FCN synchronizes metadata, validates schemas, orchestrates governance workflows, and supports federated queries. While each participating institution retains control of its source data, the FCN provides shared registries for metadata, terminology mappings, and audit logs. This hybrid federated–centralized model allows heterogeneous systems to interoperate without transferring raw data to a single repository and ensures compliance with the National Interoperability Framework (ENI) and the technical specifications of the EHDS.

The system ingests data from 4 primary domains, including primary care (AP MADRID), hospital care (SELENE and Healthcare Information System [HCIS; DXC Technology]), population-based health registries (including the minimum basic dataset CMBD, CIBELES, and the National Statistics Institute [INE]), and medication-related datasets (eg, FARMADRID). Ingested data are harmonized and transformed into a standard semantic and syntactic format using the OMOP model [21] enabling integrated analyses across heterogeneous sources.

Although storage and computation occur within a secure regional cloud operated by Madrid Digital, governance, stewardship, and access decisions remain decentralized across institutions. This preserves institutional data sovereignty while enabling standardized, privacy-preserving access and analytics through shared coordination services. The OMOP common data model and the DAMA framework support semantic interoperability, metadata governance, and quality assurance, ensuring alignment with both national [17] and EHDS requirements [8,11].

The infrastructure is also designed in accordance with the Spanish ENS. A preliminary security impact assessment has been completed to determine the system’s classification (medium–high category), and risk-based controls are being implemented to achieve ENS certification. These include penetration testing, role-based access control, incident-response protocols, and regular third-party security audits coordinated by Madrid Digital’s information-security office.

Structured data are automatically processed and encoded using internationally recognized terminologies such as *ICD-10 (International Statistical Classification of Diseases and Related Health Problems 10th Revision)*, SNOMED-CT (Systematized Nomenclature of Medicine Clinical Terms), and LOINC (Logical Observation Identifiers, Names, and Codes). In parallel, unstructured clinical data—such as free-text notes and reports—are processed using advanced NLP and ML techniques to extract meaningful information and enrich data completeness. This dual curation process ensures that both structured and unstructured data can be leveraged for research and decision-making.

The extract-transform-load process is fully documented and version-controlled to ensure traceability of data lineage. Mapping specifications follow the OMOP model conventions, including concept coverage analysis for local codes and terminologies. Deidentification follows a standardized pipeline comprising date shifting, pseudonymization of identifiers, suppression of quasi-identifiers, and aggregation of rare categories. NLP outputs undergo automated review to prevent inadvertent reidentification. These processes comply with ISO/IEC 20889:2018 principles [20] on privacy-enhancing data deidentification.

To ensure high-quality, interoperable data across systems such as CMBD, SELENE, and FARMADRID, HealthData@MAD-R&I implements a multilayered data-quality and harmonization pipeline. This includes automated ingestion validation (format, structure, and referential integrity), rule-based cleaning to correct inconsistencies and handle missingness, and semantic normalization using controlled vocabularies. A dedicated quality registry stores metrics—completeness, consistency, conformity, timeliness, and plausibility—and ML-based anomaly detection identifies outliers or unexpected patterns. This pipeline ensures that only validated, high-quality datasets are made available within the SPE, supporting robust, reproducible analytics across all use cases.

### Area 3: Validation of the HealthData@MAD-R&I Space Through Real-World Use Cases (WPs 4-7)

The third thematic area is devoted to validating the functionality, relevance, and impact of the HealthData@MAD-R&I space through the implementation of 4 real-world, data-driven use cases. These use cases serve both as proof-of-concept and as practical demonstrations of how secondary use of health data can support clinical decision-making, improve health care outcomes, and generate evidence to guide public health policy. Each use case is implemented within a dedicated work package (WP4-WP7), allowing for domain-specific focus while ensuring methodological consistency.

### Data Analysis

All use cases follow a standardized analytical framework to ensure methodological consistency, reproducibility, and compliance with data protection and governance requirements. Data analyses are conducted within the Secure Processing Environment of the HealthData@MAD-R&I space.

Structured datasets are harmonized using the OMOP common data model and undergo preprocessing procedures, including data cleaning, normalization, and feature engineering. Datasets are partitioned into training (70%), validation (15%), and testing (15%) sets. For NLP-based analyses, unstructured clinical text is semiautomatically annotated using labeling schemes aligned with SNOMED CT entities.

Depending on the specific objectives of each use case, a range of statistical and ML methods are applied, including gradient-boosted decision trees, regularized regression models, and neural network architectures. Model development and tuning are performed using cross-validation techniques. Model performance is evaluated using standard metrics such as area under the receiver operating characteristic curve, *F*_1_-score, precision–recall metrics, and calibration curves.

Model interpretability and transparency are addressed through Shapley Additive Explanations (SHAP)–based feature attribution analyses to support clinical relevance and trustworthiness. All analytical workflows are version-controlled and monitored to ensure auditability, reproducibility, and compliance with GDPR and EHDS requirements.

WP4 aims to optimize referral pathways for patients with rheumatic and musculoskeletal diseases (RMDs). The primary objective is to develop and validate predictive tools to support early diagnosis and appropriate stratification of patients at both the primary and specialist care levels. Given the high prevalence and burden of RMDs, coupled with significant diagnostic delays and misclassifications [23-25], this use case seeks to enhance referral accuracy and reduce the time to effective treatment. By integrating real-world data from EHRs across care levels—including demographic, clinical, and treatment information—this WP will identify referral patterns and clinical markers associated with optimal care pathways. The implementation of this WP is multicentric, involving collaboration between primary care teams and several university hospitals in Madrid.

WP5 focuses on the longitudinal analysis of care pathways for women who are long-term survivors of breast cancer. Using data from the SURBCAN-Madrid cohort, this use case investigates multimorbidity, health care use, and the evolution of health outcomes among women with long-term survival (>5 years after cancer remission). The objective is to identify predictive factors associated with better outcomes and to inform personalized follow-up protocols. The study integrates data from hospital and primary care settings, as well as registries and pharmacy records, and includes both survivors of breast cancer and matched control groups. The findings will support the design of evidence-based survivorship care strategies.

WP6 is dedicated to developing a predictive model for unplanned hospitalization. Building upon earlier risk stratification efforts developed in the Infobanco project [3], this use case aims to refine and validate predictive tools that identify individuals at high risk of avoidable admissions. By integrating EHRs, medication data, and hospital activity records, this WP seeks to support clinicians with decision-making tools for proactive intervention. The model is expected to improve care coordination, optimize resource allocation, and reduce unnecessary hospitalizations.

WP7 evaluates the effectiveness of statins in the prevention of cardiovascular events among individuals aged 75 years and older with no prior cardiovascular disease. This population is underrepresented in clinical trials, and the effectiveness of statins for primary prevention in this age group remains uncertain [26]. Using linked clinical, pharmaceutical, and mortality data, this use case will assess health outcomes associated with statin use, identify subgroups that benefit most, and evaluate the cost-effectiveness of current prescribing practices. The findings will contribute to more personalized prescribing strategies and inform clinical guidelines for primary prevention of cardiovascular events in older adults.

All 4 use cases follow a common analytical and governance framework, including standardized data harmonization, secure data access, and outcome evaluation using established clinical and system-level indicators. The results will be synthesized into actionable recommendations, contributing to evidence-based clinical practice and informing policy at both regional and national levels.

### Evaluation

The evaluation framework for HealthData@MAD-R&I follows a SMART approach—specific, measurable, achievable, relevant, and time-bound—to assess the platform’s performance and impact. Indicators are structured around 3 dimensions, including data quality, research output, and health care improvement. Each includes baselines, target values, and timelines for assessment. For example, data completeness and interoperability will be measured quarterly, with a baseline of current system coverage and a target of ≥90% standardized datasets by 2026. Research indicators (eg, number of publications, funded projects) will be benchmarked annually. To assess health care impact, quasi-experimental designs such as “Interrupted Time Series” and “Difference-in-Differences analyses” will be applied to evaluate changes in outcomes attributable to the platform rather than to secular trends.

Evaluation metrics are structured into 3 primary categories, including data quality, research impact, and health care improvement. Ensuring the quality of the data available within the health data space is fundamental to its success. Key indicators in this area include the completeness of datasets—for example, measured by the percentage of missing values in critical variables—alongside the accuracy of data entries validated against reference standards. Timeliness is also evaluated by tracking the speed of data ingestion and processing, while interoperability is assessed through the number of datasets successfully converted to standard models such as OMOP and made available for use. Data usage metrics, including the number of executed queries and frequency of dataset access by researchers and health care providers, further indicate the utility and accessibility of the platform.

To measure research impact, the project will monitor the number of peer-reviewed publications, conference presentations, and technical reports that derive from analyses conducted within the data space. Additional indicators include the volume of funded research projects leveraging the platform, the establishment of new collaborations among health care institutions, academic centers, and industry, and reductions in the time required to generate actionable insights compared to conventional research methods.

Health care improvement is evaluated by analyzing the influence of data-driven insights on clinical practice and health system performance. This includes reductions in unnecessary referrals and increased diagnostic precision for RMDs, enhanced coordination and follow-up in survivorship care for patients with breast cancer, lower rates of unplanned hospitalizations, and better understanding of medication effectiveness—particularly statins—in older populations. Improvements in patient outcomes, such as earlier diagnoses, fewer complications, and increased survival rates, will serve as key indicators of impact.

In addition to performance metrics, a set of broader success indicators has been defined to evaluate the overall sustainability and systemic influence of the initiative. These include the extent to which the data space becomes a valuable resource for biomedical and clinical research, the validation and clinical implementation of predictive models and decision-support tools developed through the platform, and improvements in health care delivery driven by access to high-quality, real-world data. Evidence of enhanced care pathways, greater adoption of personalized medicine approaches, and measurable cost reductions will be crucial outcomes.

Stakeholder engagement and satisfaction will also be tracked. High levels of participation from health care providers, researchers, and patients, as well as the successful inclusion of new stakeholders over time, will indicate a dynamic and sustainable ecosystem. Compliance with data protection regulations, the absence of significant data breaches, and a demonstrable level of public trust in the platform are equally important for long-term success.

Finally, the project’s contribution to the broader data ecosystem will be assessed through indicators such as the stimulation of innovation and entrepreneurship, the emergence of new products and services, the development of revenue streams to support the sustainability of the platform, and Spain’s strengthened positioning as a European leader in secondary health data use.

Through continuous monitoring and iterative evaluation based on these indicators, HealthData@MAD-R&I aims not only to ensure internal quality improvement but also to provide a robust foundation for scaling and replicating the model across other regions and sectors.

### Ethical Considerations

The protocol was approved by the Research Ethics Committees of Hospital Universitario 12 de Octubre (applications 25/442 and 25/526), Comité de Ética de la Comunidad de Madrid (application 01/25), and Hospital Clínico San Carlos (application 24/259-E). The study complies with national and European ethical and legal standards, including the GDPR (EU [European Union] 2016/679) and the European Health Data Space Regulation (EU 2025/327).

## Results

As of November 2025, the HealthData@MAD-R&I project, funded on November 1, 2024, has achieved 3 major milestones: (1) the drafting of a comprehensive data governance model that articulates principles of quality, transparency, and regulatory compliance; (2) the development of a secure, interoperable technological architecture with data federation capabilities, based on international standards such as DAMA and OMOP and incorporating artificial intelligence, ML, and NLP tools; and (3) the design and implementation of 4 real-world use cases—optimization of rheumatology referrals, follow-up of long-term survivors of breast cancer, prediction of unplanned hospitalizations, and evaluation of statin effectiveness in older adults—which will validate the health data space while addressing clinical and policy challenges. Together, these milestones demonstrate the project’s progress toward creating a sustainable, evidence-driven regional health data infrastructure.

## Discussion

The HealthData@MAD-R&I project constitutes a significant step forward in the development of health data spaces for secondary use in Europe. By establishing a federated, secure, and ethically governed data infrastructure in the Madrid region, this initiative aligns with the strategic objectives of the EHDS [8] and contributes to the creation of a harmonized framework for responsible data sharing. Its primary innovation lies in improving the usability of real-world health data—originally collected for clinical care—for purposes of research, innovation, and health system strengthening, while maintaining compliance with both Spanish and European regulatory frameworks.

A core contribution of HealthData@MAD-R&I is the development of a robust data governance model, which defines the roles and responsibilities of health data holders, access bodies, and users. This governance framework introduces oversight mechanisms to ensure transparency, equity, and security in data access and usage. By applying privacy-preserving techniques and reinforcing ethical standards, the initiative lays the groundwork for building public trust, an essential factor for the success of any secondary use health data initiative [4,10,27].

Equally important is the project’s use of advanced data harmonization processes, based on OMOP interoperability standards, which enable the transformation of heterogeneous datasets into a common semantic and syntactic structure [21,28]. This allows for scalable, reproducible analysis across care settings and institutions, addressing one of the most pressing barriers to the secondary use of health data fragmentation. Furthermore, by validating its architecture through 4 real-world use cases—covering referral optimization, survivorship care, hospitalization prediction, and medication effectiveness—the project demonstrates not only technical feasibility but also practical value in improving health care delivery.

Within the EHDS framework, HealthData@MAD-R&I is positioned primarily as a regional health data holder that provides secure and interoperable SPEs for authorized research and innovation activities. It will interoperate with national and EU-level Health Data Access Bodies through standardized metadata exchange and harmonized governance procedures. The infrastructure operates under the authority of the SERMAS), with governance jointly coordinated by the General Directorate of Health Research and teaching (DGID) and the DGSD. This structure ensures alignment with regional and European regulatory requirements while maintaining institutional autonomy and accountability.

The design and governance model of HealthData@MAD-R&I were informed by an analysis of other regional health data infrastructures in Spain, including BARDENA (Aragón), PADRIS and SIDIAP (Catalonia), BIGAN (Basque Country), VALMED (Valencia), and MED-P (Andalusia). While these initiatives have advanced secondary use of health data within their respective regions, HealthData@MAD-R&I introduces a stronger integration of governance, interoperability, and real-world validation through use cases. Its hybrid federated–centralized architecture is specifically designed to maximize scalability, reproducibility, and alignment with the EHDS regulatory framework [8], while preserving regional data sovereignty and institutional control over datasets.

Building on these regional contributions, HealthData@MAD-R&I is also positioned within a broader European context, where health data spaces are increasingly recognized as key enablers of research, innovation, and digital transformation. The EHDS provides the regulatory and technical framework to facilitate data access while safeguarding individual rights [7].

Health data spaces are expected to accelerate scientific discoveries, support evidence-based policymaking, and enhance public health preparedness [7,14]. The COVID-19 pandemic demonstrated the need for structured, interoperable, and cross-border data systems to enable real-time decision-making. Beyond crisis response, secondary health data can drive personalized medicine, improve population health surveillance, and stimulate economic growth through public–private partnerships and digital innovation [12,27].

Looking ahead, the project anticipates compliance with the wider EU digital and data governance framework, including the Data Act, Data Governance Act, Artificial Intelligence Act, Medical Devices Regulation, and Health Technology Assessment Regulation. Alignment with these instruments will ensure that the infrastructure not only supports lawful and ethical data use but also provides a foundation for trustworthy AI, transparent algorithmic evaluation, and interoperability with future cross-border health data services under the EHDS.

At the same time, these opportunities are accompanied by significant ethical, legal, and technical challenges. Privacy and data protection concerns remain at the forefront. Although regulations such as the European Union’s GDPR provide legal safeguards, ambiguities persist around the practical application of these principles within health data spaces [7]. The potential for reidentification of anonymized data, lack of explicit consent mechanisms, and growing citizen concerns about surveillance or commercial exploitation may undermine public trust. In parallel, issues of transparency in data governance—especially regarding access by private entities—raise concerns about fairness, accountability, and societal benefit [29].

Technical interoperability is another persistent barrier, particularly in countries with fragmented health care IT systems. Despite the adoption of international standards like FHIR and OMOP, differences in data quality, structure, and availability continue to hinder seamless integration. Furthermore, the governance and operational roles of health data access bodies require further clarification and consistency across jurisdictions to ensure equitable access and regulatory coherence.

Within this evolving ecosystem, HealthData@MAD-R&I demonstrates several strengths. Its federated architecture allows data to remain under the control of health institutions while enabling secure and efficient secondary access. The project integrates a wide range of clinical and administrative datasets across care levels and leverages AI and ML to unlock clinically relevant insights. Its validation through real-world use cases ensures direct applicability to pressing health challenges and facilitates the development of data-driven interventions that improve patient outcomes. The project’s alignment with national digital transformation policies and its compatibility with EHDS standards make it a scalable model for replication.

Nonetheless, the project is not without limitations. While federated systems enhance privacy, they require significant investment in infrastructure, standardization, and coordination. The heterogeneity of electronic health records and clinical terminologies in the Madrid region remains a challenge despite the harmonization efforts. Moreover, although the governance model is designed to ensure transparency, the use of opt-out consent mechanisms may provoke resistance from patients and clinicians who are concerned about autonomy and data misuse [1,29,30]. Finally, the risk of disproportionate benefit by private actors must be actively managed to preserve equity and public trust.

In conclusion, HealthData@MAD-R&I offers a pioneering approach to unlocking the potential of secondary health data for research, clinical decision-making, and public health. Its integrated governance, advanced technical architecture, and validation through impactful use cases position Madrid as a leader in digital health innovation. The long-term success of the project will depend on its ability to address persistent challenges related to interoperability, ethical governance, and public engagement. If these are met, the initiative can serve as a blueprint for other regions aiming to build transparent, secure, and effective health data ecosystems across Europe.

## Abbreviations

AI: artificial intelligence
CMBD: Conjunto Mínimo Básico de Datos
DAMA: Data Management Association
DGID: General Directorate of Health Research and Teaching
DGSD: General Directorate of Digital Health
DPIA: Data Protection Impact Assessment
EHDS: European Health Data Space
EHR: Electronic Health Record
ENI: National Interoperability Framework
ENS: National Security Framework
EU: European Union
FCN: Federated Coordination Node
FHIR: Fast Healthcare Interoperability Resources
GDPR: General Data Protection Regulation
HCIS: Healthcare Information System
HL7: Health Level 7
ICD-10: International Statistical Classification of Diseases and Related Health Problems 10th Revision
IEC: International Electrotechnical Commission
INE: National Institute of Statistics
ISO: International Organization for Standardization
LOINC: Logical Observation Identifiers, Names, and Codes
ML: machine learning
NLP: natural language processing
OMOP: Observational Medical Outcomes Partnership
RMD: rheumatologic musculoskeletal disease
SERMAS: Madrid Health Service (Servicio Madrileño de Salud)
SHAP: Shapley Additive Explanations
SNOMED cT: Systematized Nomenclature of Medicine Clinical Terms
SPE: secure processing environment
WP: work package

## Data Availability

Data sharing is restricted while the HealthData@MAD-R&I infrastructure remains under development. However, metadata, project documentation, and public deliverables are available through the RIMASalud repository. Upon completion of the project, additional non-sensitive materials, including technical documentation and example scripts, will also be deposited in RIMASalud for open access. Requests for access to restricted datasets can be submitted through the governance procedures of the Madrid Regional Health System (SERMAS) once the Secure Processing Environments become operational.
