# Correction: HealthData@MAD-R&I: Protocol for Design and Development of a Regional Health Data Infrastructure to Enable Secondary Use of Health Data in Research and Innovation

**DOI:** 10.2196/99013

**Published:** 2026-06-30

**Authors:** Montserrat León-García, Sergio Álvarez-Pérez, Janire Gesto-Gómez, Clara Urbano-Molina, Sonia Soto-Díaz, Juan Cárdenas-Valladolid, Luis Rodríguez-Rodríguez, Antonio Díaz-Holgado, Isabel del Cura-González, Javier De La Cruz-Bertolo, Noelia García-Barrio, Juan Luis Cruz-Bermúdez, Cristina García-Fernández, Carlos Rodríguez-Antolín, Laila García-Aldars, Elsa María Moreda-Sánchez, Álvaro Roldán López, José María Veganzones Alonso-Cortés, Ana Isabel Gonzalez Gonzalez, Miguel A Salinero-Fort, HealthData@MAD-R&I Working Group

**Affiliations:** 1 Fundación para la Investigación e Innovación Biosanitaria de Atención Primaria (FIIBAP) Madrid, Madrid Spain; 2 Dirección General de Investigación y Docencia Consejería de Sanidad de la Comunidad de Madrid Madrid, Madrid Spain; 3 Universidad Alfonso X el Sabio Madrid, Madrid Spain; 4 Frailty, Multimorbidity Patterns and Mortality in the Elderly Population Residing in the Community Hospital La Paz Institute for Health Research (IdiPAZ) Madrid, Madrid Spain; 5 Grupo de Patología Musculoesquelética, Fundación para la Investigación Biomédica del Hospital Clínico San Carlos Instituto de Investigación Sanitaria San Carlos (IdISSC) Madrid, Madrid Spain; 6 Gerencia Asistencial de Atención Primaria, Servicio Madrileño de Salud, Consejería de Sanidad de la Comunidad de Madrid Madrid, Madrid Spain; 7 Departamento de Especialidades Médicas y Salud Pública Universidad Rey Juan Carlos Madrid, Madrid Spain; 8 Network for Research on Chronic Diseases, Primary Care, and Health Promotion (RICAPPS) Madrid, Madrid Spain; 9 Instituto de Investigación Sanitaria Gregorio Marañón Madrid, Madrid Spain; 10 Ageing research center Karolinska Institute Solna, Stockholm Sweden; 11 Grupo de Investigación e Innovación en Transformación Digital e Ingeniería Biomédica Hospital Universitario 12 de Octubre Instituto de Investigación Sanitaria Hospital 12 de Octubre (imas12) Madrid, Madrid Spain; 12 Dirección General de Salud Digital Consejería de Digitalización de la Comunidad de Madrid Madrid, Madrid Spain; 13 Instituto de Genética Médica y Molecular Instituto de Investigación Sanitaria del Hospital Universitario La Paz Madrid, Madrid Spain; 14 See Acknowledgments

In “HealthData@MAD-R&I: Protocol for Design and Development of a Regional Health Data Infrastructure to Enable Secondary Use of Health Data in Research and Innovation” [1], the authors made one correction. The following sentence was removed from the Funding section:

This project has received funding from Horizon Europe programme under grant agreement no. 101080711 (SPIDERR).

The correction will appear in the online version of the paper on the JMIR Publications website, together with the publication of this correction notice. Because this was made after submission to PubMed, PubMed Central, and other full-text repositories, the corrected article has also been resubmitted to those repositories.
